# Computed tomography imaging features of malignant ‘triton’ tumor to facilitate its clinical diagnosis: report of two cases

**DOI:** 10.1186/s12880-022-00848-9

**Published:** 2022-07-14

**Authors:** Yuan Li, Qiling Peng, Ning Jiang, David P. Molloy, Chun Zeng, Qingchen Wu

**Affiliations:** 1 Department of Thoracic Surgery, National Cancer Center/National Clinical Research Center for Cancer/Cancer Hospital, Chinese Academy of Medical Sciences and Peking Union Medical College, Beijing, 100010 China; 2 Department of Cardiothoracic Surgery, The First Affiliated Hospital of Chongqing Medical University, No. 1 Youyi Road, Yuzhong District, Chongqing, 400016 China; 3 School of Basic Medical Science, Chongqing Medical University, Chongqing, 400016 China; 4 Department of Pathology, Chongqing Medical University, Chongqing, 400016 China; 5 Department of Radiology, The First Affiliated Hospital of Chongqing Medical University, No. 1 Youyi Road, Yuzhong District, Chongqing, 400016 China

**Keywords:** Computed tomography, Malignant triton tumor, Presumptive diagnosis, Case report

## Abstract

**Background:**

Malignant ‘triton’ tumor is an extremely rare subtype of malignant periphery nerve sheath tumors. Clinical diagnosis of malignant triton tumor is difficult before surgery due to its low incidence and the lack of knowledge. Therefore, to describe and summarize the CT imaging characteristics of malignant triton tumor is of great assistance for early and preoperative diagnosis.

**Case presentation:**

Two cases suspected of MTT by CT scan before operation were closely observed. The diagnosis of malignant triton tumor was eventually confirmed by immunochemical assay, which verified speculation of CT scans. Huge, irregular, well-circumscribed lobulated mass-like shadows can be observed from these patients by CT scans. Besides, heterogeneity of density within the body of tumor was well-established by CT scans, together with linear septum. Meanwhile, CT scans demonstrated that calcifications were remarkable at the margin of tumor body.

**Conclusions:**

Some CT image features from two cases were presented as a reference for the preoperative consideration of MTT: (i) enormity of mass-like shadow; (ii) presence of well-circumscribed lobulated shape; (iii) septum within the well-defined mass accompanied with hemorrhage, necrosis and cystic changes as well as calcification, especially within neurofibromatosis type 1 patients.

**Supplementary Information:**

The online version contains supplementary material available at 10.1186/s12880-022-00848-9.

## Background

Malignant schwannomas with rhabdomyosarcomatous differentiation, originally described by Masson [1] and now designated malignant ‘Triton’ tumor (MTT), appear as neurogenic sarcomas with skeletal muscle differentiation in squamous epithelium, bone and possibly fat tissues [2]. MTT is an extremely rare kind of malignant periphery nerve sheath tumors (MPNSTs), with a worldwide incidence of fewer than 200 cases reported to date [3]. Of these, 69% of MTT have been diagnosed in young male patients with neurofibromatosis type 1 (NF-1) and 31% are sporadic cases mostly occurring in older women [4, 5]. At present, treatment scenarios for MTT include radical surgical resection, adjuvant chemotherapy and radiation, with the latter consideration as the standard protocol for MPNSTs or soft tissue sarcoma [6, 7]. With respect of tumor location, MTT primarily arise in head, neck and trunk regions, but also infrequently observed in lung, mediastinum, abdomen and prostate tissues [8, 9], which invariably culminates with the untimely demise of the infected individual.

Recurrence of MTT after excision is common and with a 5-year survival rate of merely 12%, prognosis remains extremely unfavorable for afflicted individuals [4, 6, 10]. Moreover, in the absence of suitable awareness and education concerning a disease of intrinsically low incidence within the general human population, pre-surgical misdiagnoses of MTT are frequent. Early identification and analysis of the clinic pathology of MTT is, therefore, of critical importance. The first truly indicative criteria for a positive diagnosis of tumorigenic MTT was derived in 1973 and include location along a peripheral nerve ganglioneuroma, Schwann cell tumor characteristics and observation of rhabdomyoblasts within the body of tumor [11]. Subsequent immunohistochemical (IHC) analyses for the skeletal muscle-specific markers myogenin or Myo-D1 are then used as confirmation of malignancy [9]. Unfortunately, assays for the presence of rhabdomyoblasts with MTT are prone to error being reliant upon objective separation of MTT with sample that contain significant numbers of Schwann cell that negatively stain for Desmin and myogenin and/or Myo-D1 [2]. Such investigations are further impeded as IHC assessments are performed post-surgery, although pathological evaluation still provides a reliable tool for neurogenic tumor classification.

Until recently, computed tomography (CT) scan is used in preoperative consideration of MTT. This enables discernment of anatomical features to be made with high precision leading to increased accuracy of consideration, staging and monitoring of morphological changes in MTT during preoperative periods. Overall, the technique is a noninvasive pre-surgical alternative for diagnosis of MTT that ultimately allows for improvements in both palliative care and longevity of patients.

## Case presentation

Two patients were closely observed in our hospital and diagnosed as MTT by pathology after surgery. The definite diagnosis of MTT for both cases was established on following morphologic grounds and immunochemistry [3, 12]: malignant peripheral nerve tumor is main component, within where rhabdomyosarcoma components scattered; simultaneous positivity of IHC including S-100, Desmin and Myo-D1. The informed consent has been obtained from both patients and their immediate families to publish these details in this paper.

### Case 1

The first case was a 41-year-old female who presented with progressively worsening chest tightness and shortness of breath for more than 2 months prior to admission. Contrast-enhanced chest CT revealed a mass in the medial basal segment of the right lower lobe. Then, right lower lobectomy was performed after relevant preoperative examination was completed. Postoperative pathological diagnosis was improved by IHC. The IHC assay was positive for S-100 protein, Myo-D1 and Desmin and when combined, these findings supported a diagnosis of MTT. Pathological descriptions and related figures were presented in Additional file 1: Document S1.

**Fig. 1:**
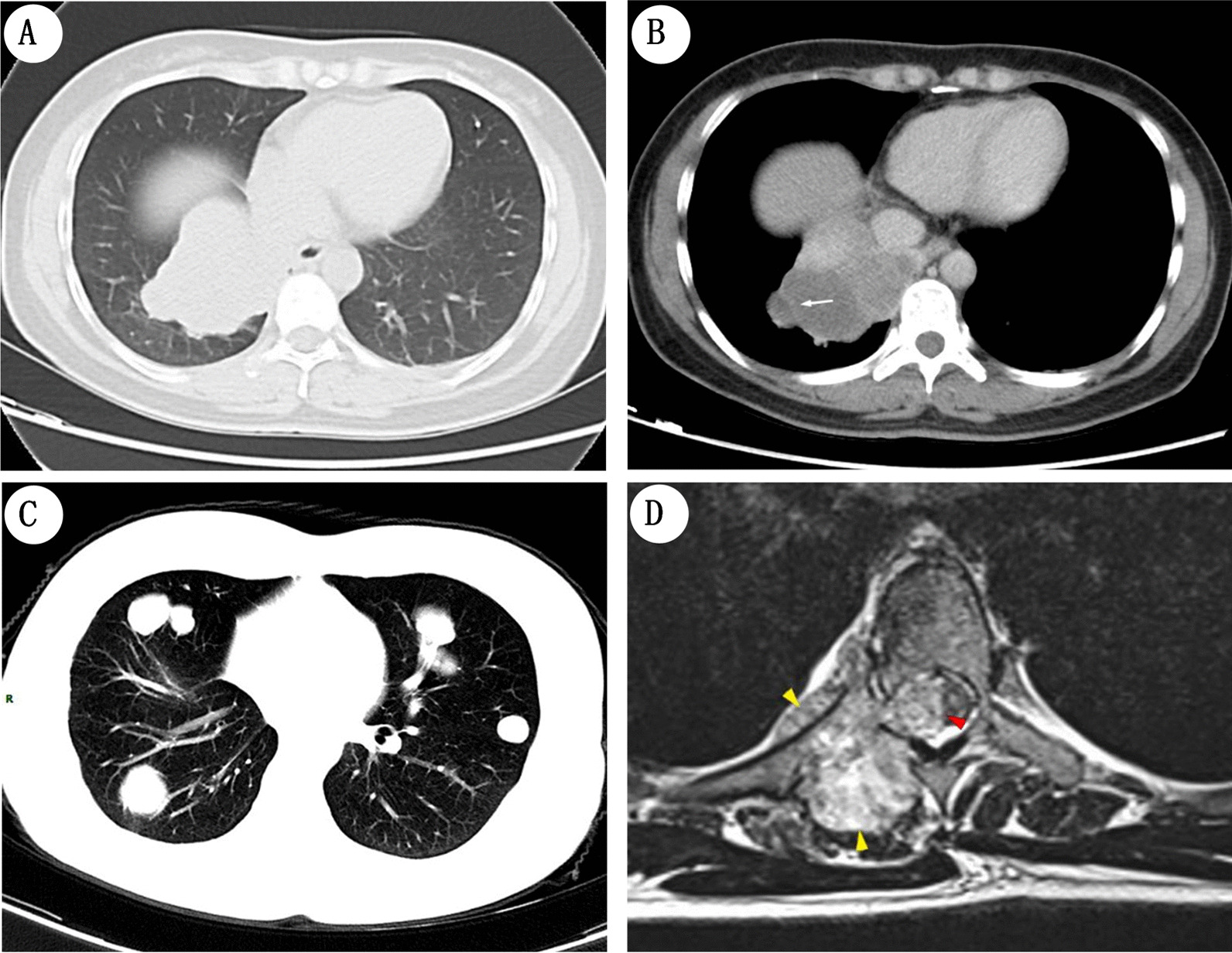
CT and MRI images of the 41-year-old female patient. **A** Chest CT images shows that a huge irregular and well-defined mass in medial basal segment of lower lobe of right lung. **B** Contrast-enhanced chest CT scan in delayed phase illustrates a mass-like shadow, which has irregular and well-circumscribed lobulated shape. Low-density cystic mass is obvious at the margin of the lesion (*white arrow*). **C** Five months later, chest CT scan displays multiple well-defined nodules and masses in bilateral lungs, suggesting recurrence and metastasis of MTT. **D** MRI image indicates that a tissue mass with homogeneous density grows in the vertebral index at T4-T5. Rather more radical anatomical consequences of MTT metastasis within thoracic region were also noted and included severe destruction of bone tissue on the right thoracic pedicle, plate and transverse processes of the T4 and T5 segments alongside tumor invasion of adjacent ribs, pleura and soft tissues (*yellow arrow*s). Aside these, an outward dispersal of the lesion from the spinal dura mater and uncontrolled growth within the thoracic spinal cord was also observed (*red arrow*)

**Fig. 2:**
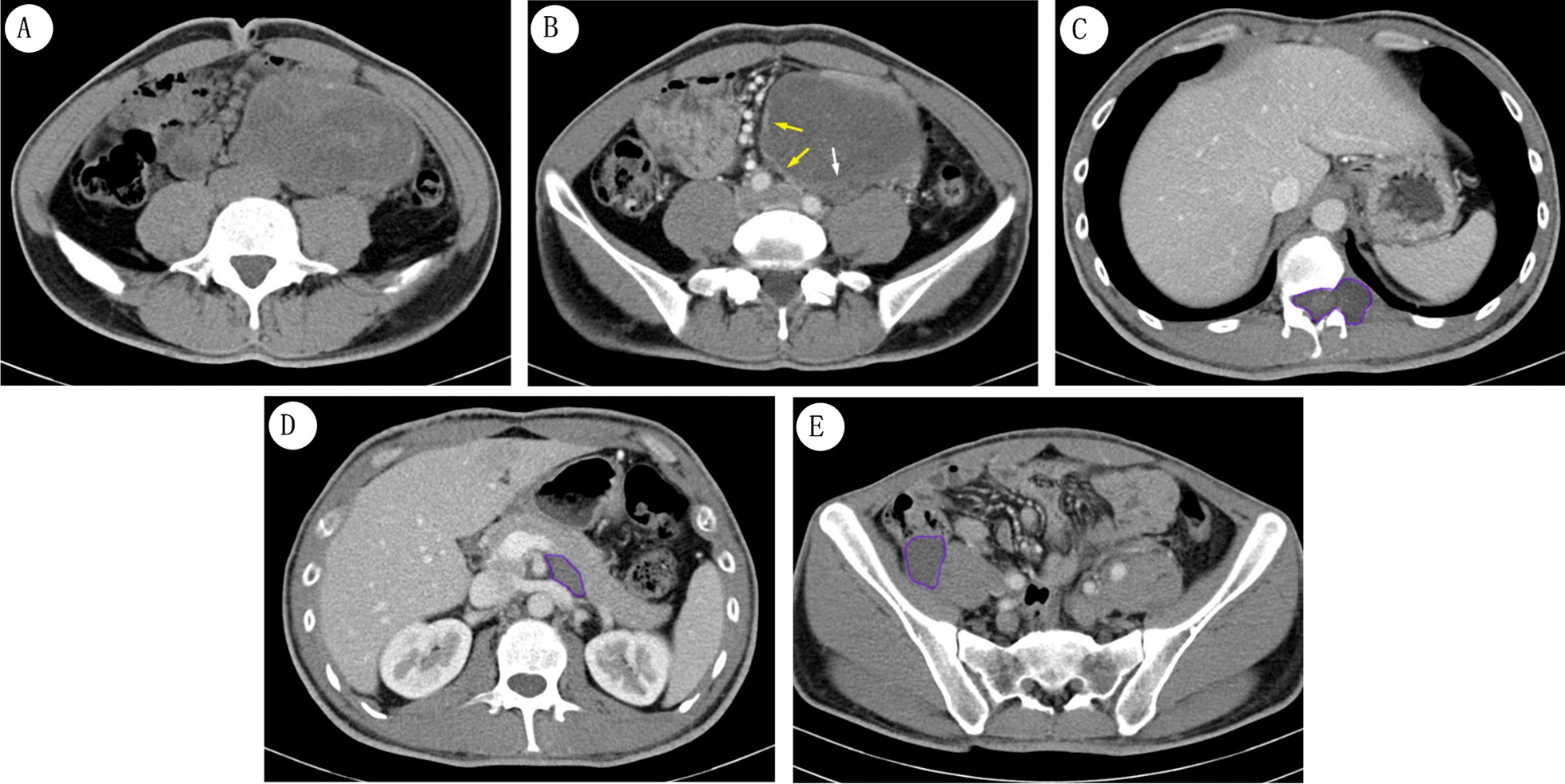
CT images of the 37-year-old male patient with a family history of NF-1. **A** The abdominal CT scan shows an enormous mass was in abdominal cavity. It is obvious that calcifications have occurred at the margin of mass shadow. **B** By contrast-enhanced CT scan, linear septum (*white arrow*) and partial edge (*yellow arrows*) are clear. In addition, A few hypodense nodules (*purple circles*) are located between the T12 and L1 (**C**), behind the pancreas body (**D**) and beside the right iliopsoas muscle (**E**)

Initially, preoperative chest CT scan revealed a large non-MPNST/soft tissue sarcoma mass of 7.7 cm × 6.0 cm, located at the medial base of the lower lobe of the right lung (Fig. 1A). Additional imaging analyses obtained using contrast-enhanced CT scans of the thoracic region re-affirmed the presence of an irregular hemorrhagic or necrotic, well-circumscribed lobule (Fig. 1B). A low-density cyst was also evidenced by absence of mediastinal and hilar node enlargements in the surrounding region and a distinct septate demarcation within the cellular mass at the lesion periphery (Fig. 1B). The patient did not undergo any postoperative chemotherapy or radiotherapy. Five months after surgery, the patient seemed to have fully recovered, with only mild refractory dyskinesia, limb numbness, back pain and dysuria. During this period, long-term prognosis appeared to be positive, as postoperative re-examination of the chest area in CT scans revealed no recurrence of the mass-like shadow within the operative region. However, several well-defined and differentially sized homogeneous nodules were seen as persistent within the bilateral lungs which raised concerns of MTT metastasis and possible malignancy (Fig. 1C). To find out discomfort arising from clinical symptoms, further investigations were performed through magnetic resonance imaging (MRI) upon the spinal cord. As shown in MRI image, a homogeneous tissue mass was observed to be expanding within the vertebral index at the 4th and 5th thoracic (T4–T5) segments causing microbleeds (Fig. 1D). Rather more radical anatomical consequences of MTT metastasis within thoracic region were also noted and included severe destruction of bone tissue on the right thoracic pedicle, plate and transverse processes of the T4 and T5 segments alongside tumor invasion of adjacent ribs, pleura and soft tissues (yellow arrows in Fig. 1D). Aside these, an outward dispersal of the lesion from the spinal dura mater and uncontrolled growth within the thoracic spinal cord were also observed (red arrow in Fig. 2D). Unfortunately, these symptoms were indicative of rapid multiple distal metastasis of MTT and the patient passed away seven months after surgery.

### Case 2

The second patient was a 37-year-old male with dull pain in the left lower quadrant for 2 months. There was a paternal familial predisposition of NF-1 inclusive of his uncle and grandfather. CT scanning of the abdominal region revealed the presence of a heterogeneous hemorrhagic or necrotic discoid mass 10 cm × 6.3 cm in size located within the left-side abdominal cavity (Fig. 2A). Calcifications were also evidenced in CT scans occurring at the margin of the mass. In contrast-enhanced CT scans, the margin of the mass was more prominent within the patient’s chest region (Fig. 2B) and similarities to the aforementioned *case 1* with the presence of a linear septum within the cellular mass were noted (white arrow in Fig. 2B). Furthermore, an irregular hypodense nodule was observed in between the T12 and the 1st lumbar (L1) vertebral canal interspersed with the left foramen intervertebral disc of the spinal cord (Fig. 2C). The lesion also resulted in significant compression of the adjacent vertebral bodies and adnexal bones and nodule elongation. Similar nodules also appeared posterior to the pancreas body and proximal to the right iliopsoas muscle and abdominal aortic branch (Fig. 2D, E). Originally, the mass was deemed to be a neurofibroma. However, additional pathological analysis and positives for Desmin, Myo-D1 and S-100 protein IHCs were more consistent with a diagnosis of MTT.

**Fig. 3:**
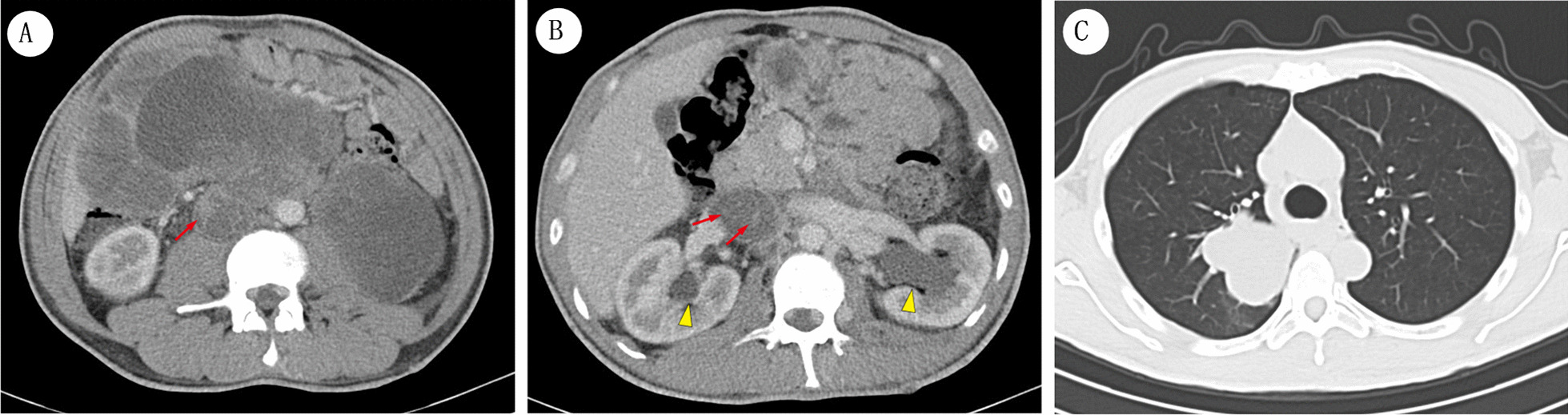
CT images of the 37-year-old male, five months later after radical mass excision. **A** Contrast-enhanced abdominal CT scan shows multiple irregular nodules and masses in abdominal cavity and retroperitoneal spaces. Tumor embolus was distinct in postcava (*red arrows* in **A** and **B**). **B** It is observed that reappeared mass grows into the perirenal spaces and blocks ureters on both sides, which give rise to pelvicalyceal dilatation and hydronephrosis (*yellow arrows*). **C** Chest CT scan demonstrates a huge well-defined mass appears adjacent to the right-side pulmonary hilum

This patient strongly refused postoperative chemotherapy and radiotherapy and developed progressive dysuria 5 months after surgery. Upon re-examination of the chest and abdominal regions by CT scans, multiple irregular nodules and masses within the abdominal cavity and retroperitoneal space were observed, consistent with a diagnosis of the recurrence of MTT (Fig. 3A). Once again, it was apparent that changes in cyst morphology, necrosis, hemorrhage and presence of septate lesions were, therefore, characteristics unique to MTT tumorigenesis. We noted that resurgence of tissue mass was accompanied by invasion of the perirenal spaces and obstruction of both ureters resulting in pelvicalyceal dilatation and hydronephrosis by enlargement of nodules in the perinephric space (Fig. 3B). Tumor embolus features in bilateral iliac veins, right atrium and postcava (Fig. 3A, B) were also observed. Meanwhile, in contrast-enhanced CT scans of the chest area, a large well-defined lobulated mass, 5.2 cm × 3.2 cm, occurred adjacent to the right-side pulmonary hilum (Fig. 3C). By contrast, the hypodense nodules which were located between the T12 and L1, behind the pancreas body and beside the right iliopsoas muscle remained unchanged from preoperative conditions (Fig. 2C–E). The patient was subsequently discharged, and the case followed up with a clinical study.

## Discussion and conclusions

MTT accounts for 5% among MPNSTs which display different patterns of malignancy in areas of mesenchymal differentiation [3, 13]. Of these, two subcategories are further refined according to clinical status of associated NF-1 [14, 15]. To date, definition of MTT etiology, pathogenesis and genetics is imprecise. And identification of clinical symptoms is somewhat subjective depending upon the location, size and presence or absence of nerve compression and adjacent tissue metastasis. Prognosis for many MTT sufferers is certainly less favorable than for more classical MPNSTs especially, where a correct diagnosis must be accurately informed from familial and clinical history and knowledge of the complexities of individual MTT subtypes. Notably, a definitive diagnosis for an MTT is based upon observations from histological staining and true positives for S-100 protein, Myo-D1 and Desmin in post-surgical IHC assays [8, 9]. Clinical histories of most patients with MTT cases typically range between several months to more than ten years in all age groups [16, 17]. In this report, the two patients were young and of similar age, 37 and 41 years old, respectively. And MTT arose in rare locations of lung and abdomen in both cases. It was noted that diagnosis of MTT in this young male patient (*Case 2*) was also assisted by his familial predisposition of NF-1, which was absent within the female (*Case 1*).

Diagnosis of MTT is difficult prior to surgery, and recent occurrence where imaging analyses were applied for reporting of the clinical manifestations of MTT are scarce [18, 19], especially for MTT versus other traditional MPNSTs. Owing to the diversity of these tumors, image features tend to be non-specific and low in descriptive clarity. Both present as a large soft tissue mass on image examination and conventional MPNSTs are also generally heterogeneous. Other image features in common include ill-defined margin, intratumoral lobulation, peritumoral edema, calcification lesions and destruction of adjacent bone tissues. However, MTT presented with some high-density stripe-like entities distributed throughout the mass on occasion. Unfortunately, the structural characteristics of rhabdomyosarcomas are frequently ill-defined or generally unobserved within CT images. Although, it is plausible that some CT imaging features are masked or obscured in the presence of an abundant blood supply whereby the mass of the MTT increases rapidly and disproportionally within a short time frame or in the presence of Schwann cell metaplasia of neural crest during a state high-malignancy [20]. At times, hemorrhage, necrosis and cystic morphological changes are observed in the lesions with a distinct high-density calcification shadow [21, 22]. Importantly, contrast-enhanced CT scan can mildly enhance the peripheral features of the mass, allowing the specificity of MTT grading based on the heterogeneity of the mass and the linear or annular shape of the atrial septal shadow. Meanwhile, several features of MTT was shown in MRI scan, including low-intensity masses and septal structures on T1—and T2-weighted images [1].

It is regretful that MRI is rarely used in pulmonary imaging in clinical practice and is usually performed only when schwannoma and sarcoma lesions are considered. This precisely limits the diagnosis of MTT and makes many relevant studies lack the contrast between MRI and CT images. Therefore, when doctors fail to consider this rare disease, it is very easy to miss diagnosis and delay the treatment of this highly malignant tumor. This is also the focus of this report, hoping to summarize the characteristics of MTT in CT to facilitate the clinical diagnosis of MTT. When considering the possibility of MTT, the profile of features observed on CT scans can improve accuracy and help doctors develop a comprehensive examination, diagnosis, and treatment plan.

In conclusion, although the definite diagnosis of MTT depends on pathology, imaging can help physicians avoid the omission of rare diseases such as MTT. To make clinicians associate MTT as soon as possible, we performed a detailed CT image analysis to get hints of relevant characteristics from two rare clinical cases. Here, we present the reference clinical features for the diagnosis of MTT based on CT analysis: (i) enormity of mass-like shadow; (ii) presence of well-circumscribed lobulated shape; (iii) septum within the well-defined mass accompanied with hemorrhage, necrosis and cystic changes as well as calcification, especially within NF-1 patients. While these may not be the ‘gold standard’, we wish they can serve as early references to assist doctors in considering MTT.

## Supplementary Information

**Additional file 1**. **Document S1** The MTT pathological features of hematoxylin-eosin (HE) staining in *Case 1*.

## Data Availability

The datasets used and/or analyzed during the current study are available from the corresponding author on reasonable request.

## Abbreviations

MTT: Malignant triton tumor
MPNSTs: Malignant periphery nerve sheath tumors
NF-1: Neurofibromatosis Type 1
IHC: Immunohistochemical
CT: Computed tomography
H&E: Hematoxylin and eosin
