# Supplementary material for: Computed tomography imaging features of malignant ‘triton’ tumor to facilitate its clinical diagnosis: report of two cases
Source: BMC Med Imaging. 2022 Jul 14;22:125. doi: 10.1186/s12880-022-00848-9 (PMC9284860; doi:10.1186/s12880-022-00848-9)
Supplement: Supplementary file 1 — Additional file 1. Document S1 The MTT pathological features of hematoxylin-eosin (HE) staining in Case 1. [file 12880_2022_848_MOESM1_ESM.pdf]

The mass of the 41-year-old female patient (*Case 1*) was postoperatively diagnosed as MTT. And the representative pathological features of hematoxylin-eosin (HE) staining were originally shown below. All specimens were fixed with 4% neutral formaldehyde. After dehydration in graded alcohol, the fixed tissues were embedded in paraffin, and then sliced (4μm thickness). HE staining was performed and slides were observed under optical microscope (microscope: BX43, objective lenses: plan achromat (PLN), illumination: brightfield (LED), Olympus (China) Co., Ltd.). Images were obtained by the LC35 digital microscope camera and LCmicro V2.3.1 software (Olympus (China) Co., Ltd.).

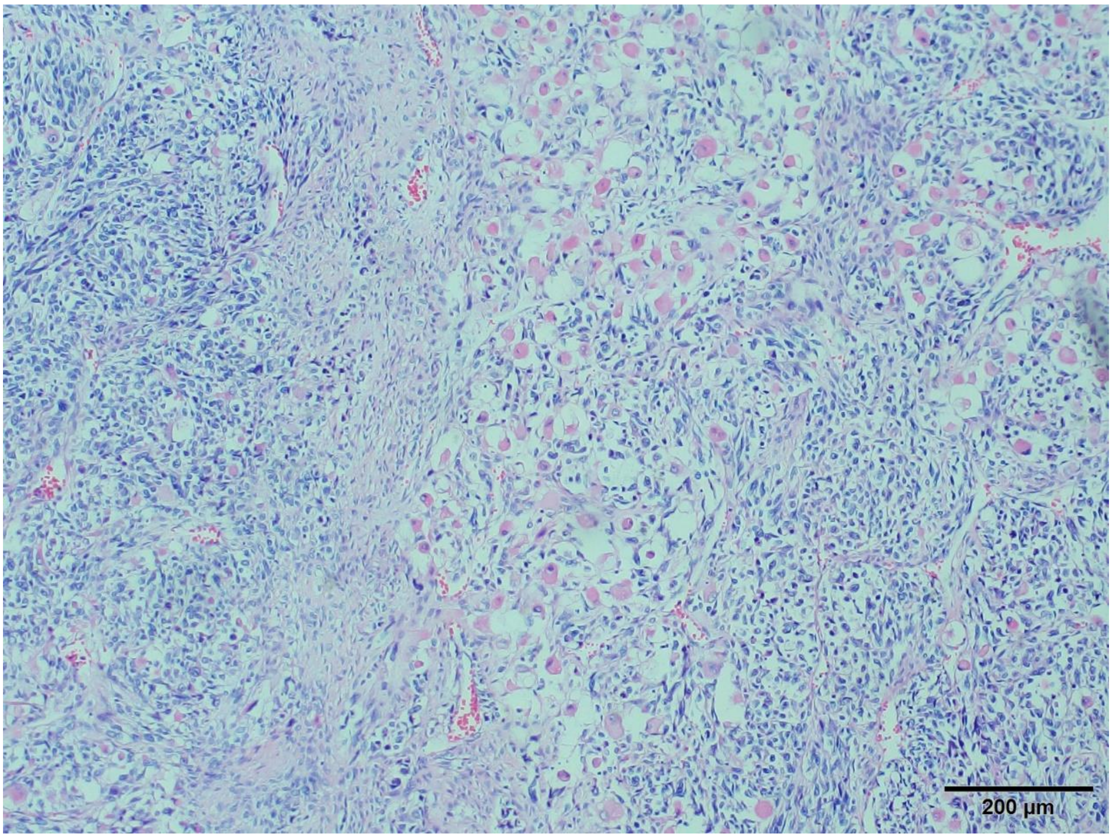

Supplemental Fig. 1 A tumorous growth was clearly identified as a discrete mass of hyperchromatic spindle cells with indistinct cytoplasm after H&E staining. Objective lens: 10× (NA 0.25).

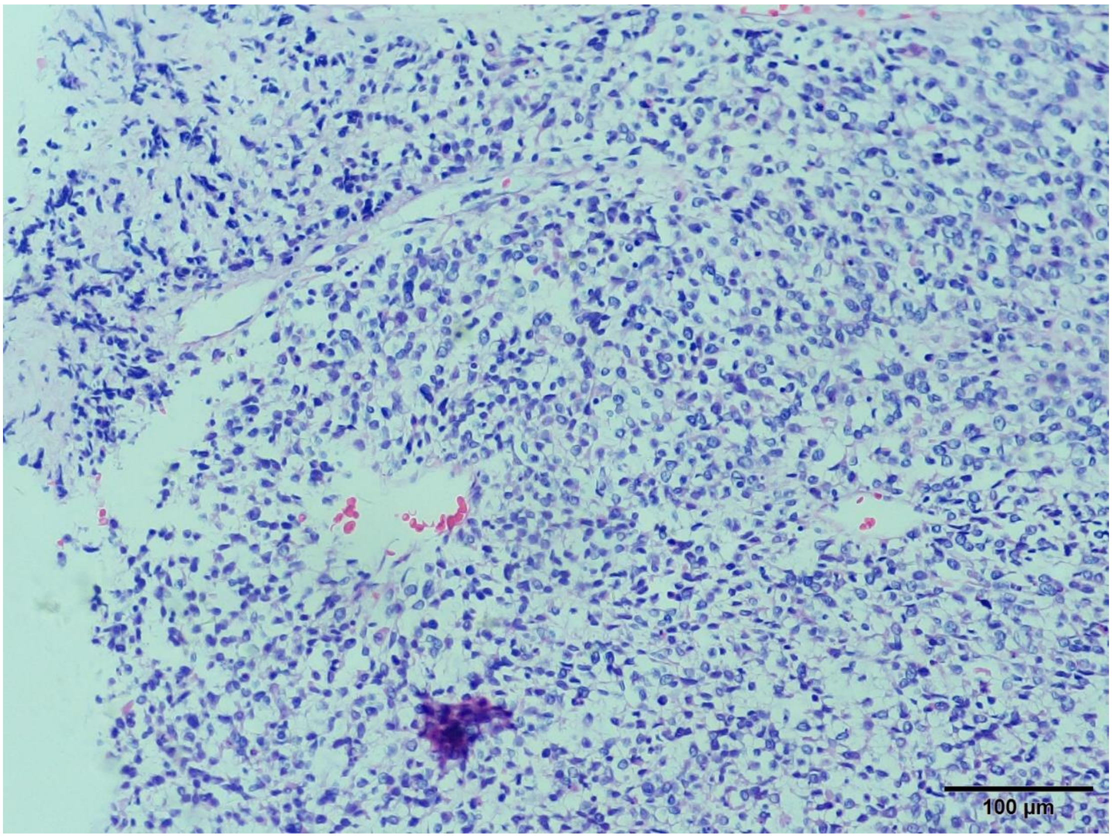

Supplemental Fig. 2 The overall architecture appeared to be either diffuse or alternate hypo-/dense-cellular regions. The tumor tissue could therefore be assigned microscopically as a malignant schwannoma of variable cellular distribution with non-homogenous rhabdomyosarcomatous features. Objective lens: 20× (NA 0.4).

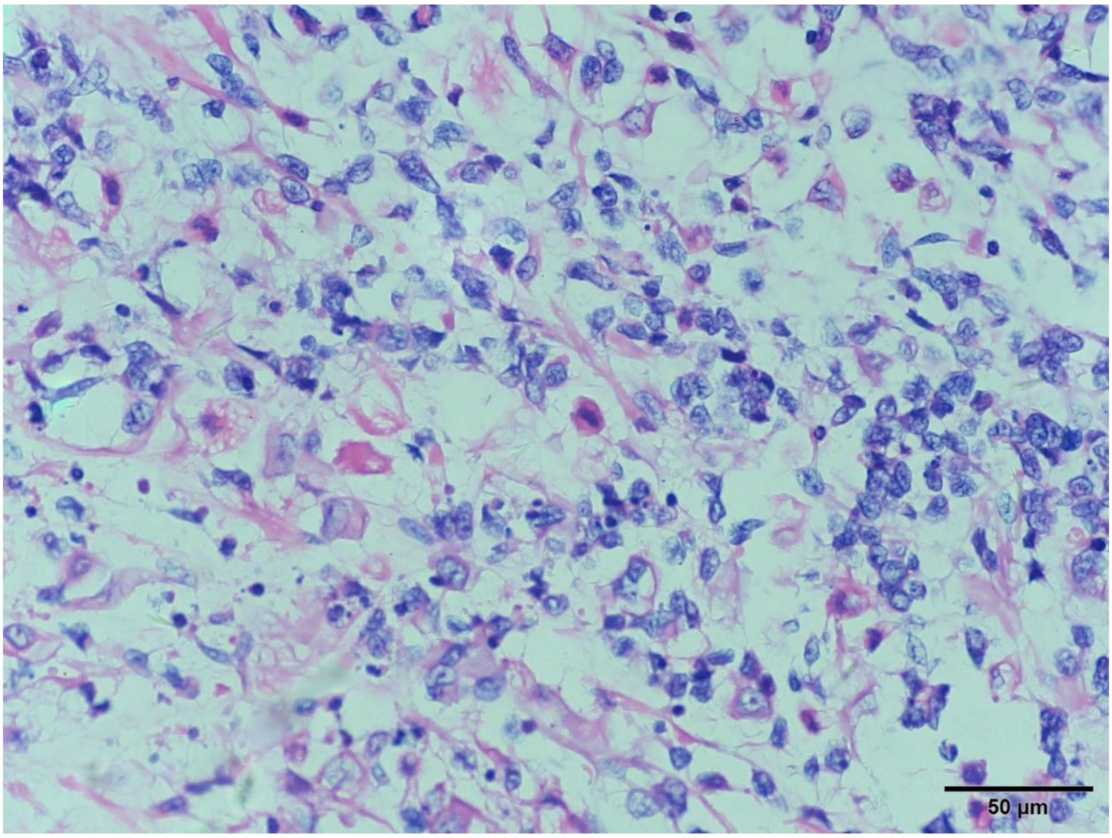

Supplemental Fig. 3 Rhabdomyoblasts with a dense eosinophilic cytoplasm and cytoplasmic cross striations were observed scattered throughout the stroma and mainly comprised of pleomorphic spindle cells. Objective lens: 40× (NA 0.65).
